# A framework for understanding, designing, developing and evaluating learning health systems

**DOI:** 10.1002/lrh2.10315

**Published:** 2022-05-20

**Authors:** Tom Foley, Luke Vale

**Affiliations:** ^1^ PI Learning Healthcare Project, Health Economics Group Population Health Sciences Institute, Newcastle University Newcastle‐upon‐Tyne UK; ^2^ Health Economics Group Population Health Sciences Institute, Newcastle University Newcastle‐upon‐Tyne UK

**Keywords:** implementation science, informatics, learning health systems, learning healthcare systems, quality improvement

## Abstract

**Introduction:**

A Learning Health System is not a technical project. It is the evolution of an existing health system into one capable of learning from every patient. This paper outlines a recently published framework intended to aid the understanding, design, development and evaluation of Learning Health Systems.

**Methods:**

This work extended an existing repository of Learning Health System evidence, adding five more workshops. The total was subjected to thematic analysis, yielding a framework of elements important to understanding, designing, developing and evaluating Learning Health Systems. Purposeful literature reviews were conducted on each element. The findings were revised following a review by a group of international experts.

**Results:**

The resulting framework was arranged around four questions:

1. What is our rationale for developing a Learning Health System?
  There can be many reasons for developing a Learning Health System. Understanding these will guide its development.
2. What sources of complexity exist at the system and the intervention level?
  An understanding of complexity is central to making Learning Health Systems work. The non‐adoption, abandonment, scale‐up, spread and sustainability framework was utilised to help understand and manage it.
3. What strategic approaches to change do we need to consider?
  A range of strategic issues must be addressed to enable successful change in a Learning Health System. These include, strategy, organisational structure, culture, workforce, implementation science, behaviour change, co‐design and evaluation.
4. What technical building blocks will we need?
  A Learning Health System must capture data from practice, turn it into knowledge and apply it back into practice. There are many methods to achieve this and a range of platforms to help.

**Discussion:**

The results form a framework for understanding, designing, developing and evaluating Learning Health Systems at any scale.

**Conclusion:**

It is hoped that this framework will evolve with use and feedback.

## 1 INTRODUCTION

The challenges facing healthcare are well rehearsed.^1^ Data and technology are often cited as the solution. However, health information technology often fails to deliver.^2^ The development and adoption of a Learning Health System has been proposed as a way of organising health systems and digital interventions to overcome previous failures.

A Learning Health System is not a new technical project within an existing health system, rather, it is the evolution of the existing system into one that is capable of learning from every patient who is treated.

The concept of a Learning Health System was introduced to healthcare in 2007 by the United States Institute of Medicine (IoM, now the National Academy of Medicine),^3^ which later defined it as a system in which:

“science, informatics, incentives, and culture are aligned for continuous improvement and innovation, with best practices seamlessly embedded in the delivery process, [with] patients and families active participants in all elements, and new knowledge captured as an integral by‐product of the delivery experience.”^4^

As awareness of the potential of Learning Health Systems has grown, a scientific ecosystem has developed around the Learning Health System concept, embodied by new university departments,^5^ conferences^6^, ^7^ and an academic journal.^8^

Several frameworks have been proposed to guide the development of Learning Health Systems.^9^, ^10^, ^11^, ^12^, ^13^, ^14^ While valuable, none of these has been universally applied in the development of new Learning Health Systems across settings and at different scales, covering social and technical factors. One has been successfully scaled across many new conditions internationally,^14^ but does not provide an organisation‐level framework. Another is very widely cited and applies at any organisational scale.^15^ However, the information available on the approach does not include detail on the full range of sociotechnical elements that often make the difference between success and failure.^9^ Other frameworks may be too high‐level to be actionable, too specific to allow local tailoring, fail to account for complexity and sociotechnical challenges or are not accessible to the general reader.

**FIGURE 1:**
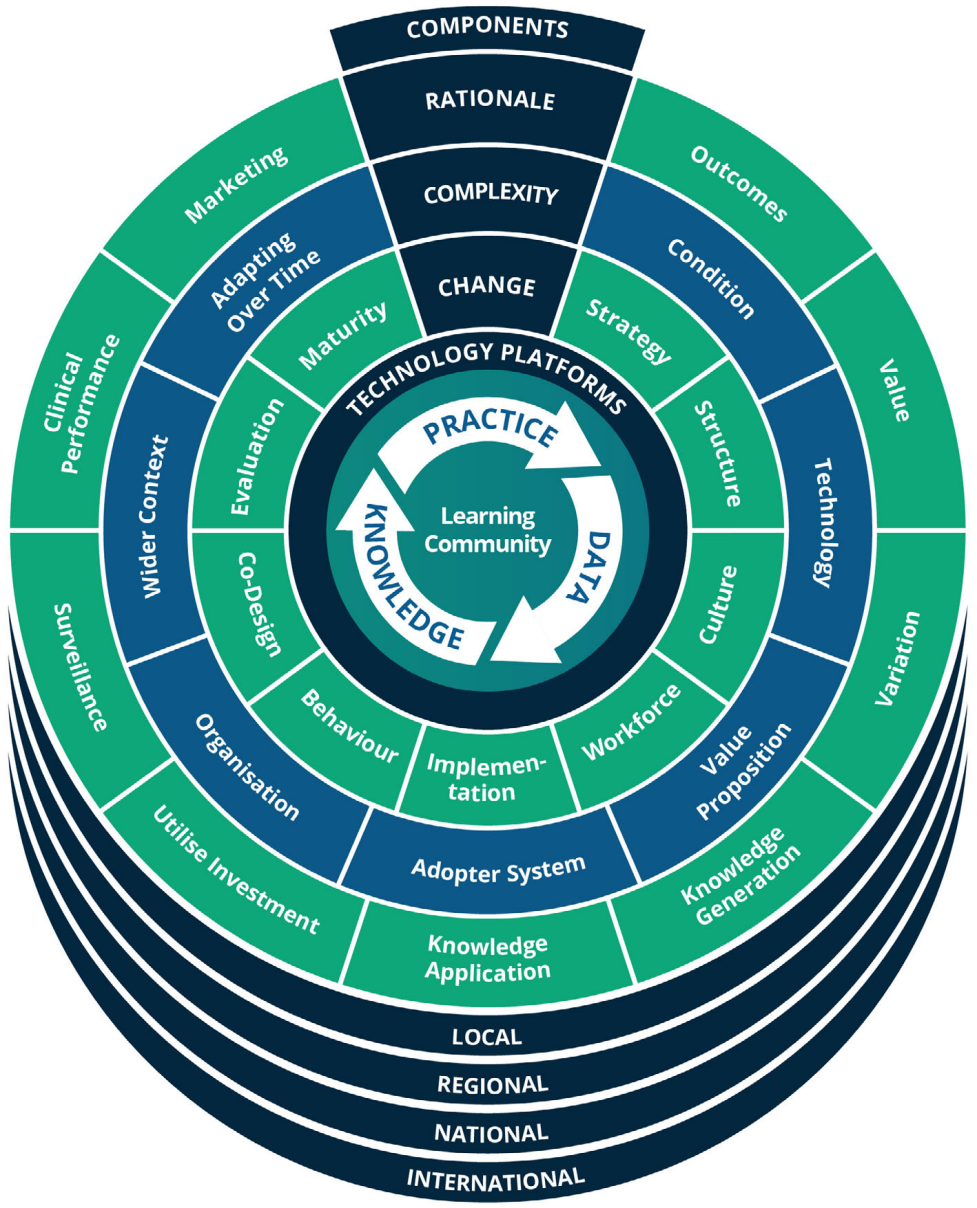
The Learning Health System Framework

This paper presents a new framework, visualised in Figure 1. This builds upon existing frameworks and aims to overcome some of the limitations noted above.

The framework described in Figure 1 was first outlined in a Health Foundation funded report in May 2021^16^ and has since been applied in a number of settings in order to aid the understanding, design, development and evaluation of Learning Health Systems. It is intended to be applicable at a local, regional, national and international scale. In the spirt of a learning system, this framework is a starting point that will evolve with use. It is hoped that this article will bring the framework to a new audience, who can participate in its evolution.

## 2 METHODS

This work builds on the 2015 report, The Potential of Learning Healthcare Systems.^17^ The report established a web‐based repository of literature, interviews, expert workshops and other material related to Learning Health Systems.^18^ Members of the public, patients and experts were invited to comment on the website material, which was then thematically analysed to produce the report. The website was updated with new findings as they emerged, even after publication of the report.

The interviews sought to explore the technical feasibility of Learning Health Systems and were conducted with international experts. Workshops focused on the, ethical, legal, regulatory, workforce planning, training, and economic implications of these potential developments. The interviews and workshops identified points around which there was consensus, disagreement or insufficient information to form an opinion.

For this current work, the structured review was updated with the addition of recently published systematic reviews on Learning Health Systems.^19^, ^20^ Interviews and workshops conducted for the previous report were supplemented with five new expert workshops, covering topics that the authors identified as being important to the success of Learning Health Systems but not comprehensively covered in the literature of that field.

The first two workshops were face‐to‐face, but the final three were conducted online, due to Covid‐19 restrictions. Each workshop was chaired by a subject matter expert and drew upon a pre‐developed topic guide. This guide was developed by the authors and workshop chair. It was intended to target gaps and prompt discussion during the workshop. During each workshop, notes of the discussion were made, along with copies of any material produced. In addition, the online workshops were recorded. From these materials, a detailed summary of each workshop was produced.

Table 1 describes the topic of each workshop, the number of participants and their backgrounds. Each topic was focused on a broad area, relevant to the design and implementation of a Learning Health System. Findings were agreed with participants and added to the project website.^21^

**TABLE 1:** Outline of workshop topics, and background of workshop participants

Workshop topic	Number of participants	Background of participants
Mobilising Computable Biomedical Knowledge	52	Industry Academia Health service organisations
Non‐adoption, Abandonment, Scale‐up, Spread and Sustainability in Learning Health Systems	15	Implementation Science Academia Industry Health sciences
Evaluation in Learning Health Systems	9	Academia Health service organisations
Participatory Co‐Design in Learning Health Systems	15	Academia Health service organisations Charities Policy research groups
Platforms in Learning Health Systems	7	Health service organisations Industry

The interviews and products of the expert workshops, contained on the project website, along with the recent literature reviews were thematically analysed to construct a framework to aid the understanding, design, development and evaluation of Learning Health Systems.

Rapid purposeful literature reviews were then conducted for each element of the framework. These aimed to provide an introduction to the topic, rather than a systematic review. The results of these activities were presented in a draft report that was circulated for unstructured peer reviewed by a group of international Learning Health System experts, who had been selected on the basis of contribution to the field and willingness to participate (see acknowledgements). The framework was revised following their expert feedback.

No formal ethical approval for this work was sought. Potential workshop participants were identified from publicly available sources and asked if they would be willing to participate. Where workshops were recorded this was done with the verbal consent of all those participating.

## 3 RESULTS

Synopses of three of the expert workshops: (1) non‐adoption, abandonment, scale‐up, spread and sustainability in Learning Health Systems; (2) platforms in Learning Health Systems: and (3) evaluation in Learning Health Systems. These workshops were uploaded to the project website^22^ alongside records of previous interviews and workshops that had been conducted since 2014. Videos of a fourth workshop (Mobilising Computable Biomedical Knowledge) have been made available^23^, ^24^, ^25^, ^26^ and documented through six independent peer reviewed articles.^27^, ^28^, ^29^, ^30^, ^31^, ^32^

The framework draws on a wide range of literature. References in this paper include 45 relevant peer reviewed articles, 58 items of grey literature and 11 links to interviews or workshops conducted by the authors.

The thematic analysis identified a large number of issues that could influence the success of a Learning Health System. These were grouped into 30 topics that each applied at multiple scales, from local, to regional, national and international. These topics were then grouped under four questions that anyone developing a Learning Health System would need to ask (Box 1). These questions would be visited in an iterative, rather than a linear fashion. Findings related to each of these are provided in the following subsections.

BOX 1 **Key questions to ask when developing a Learning Health System**

1. What is our rationale for developing a Learning Health System?
2. What sources of complexity exist at the system and the intervention level?
3. What strategic approaches to change do we need to consider?
4. What technical building blocks will we need?

### 3.1 What is our rationale for developing a Learning Health System?

**FIGURE 2:**
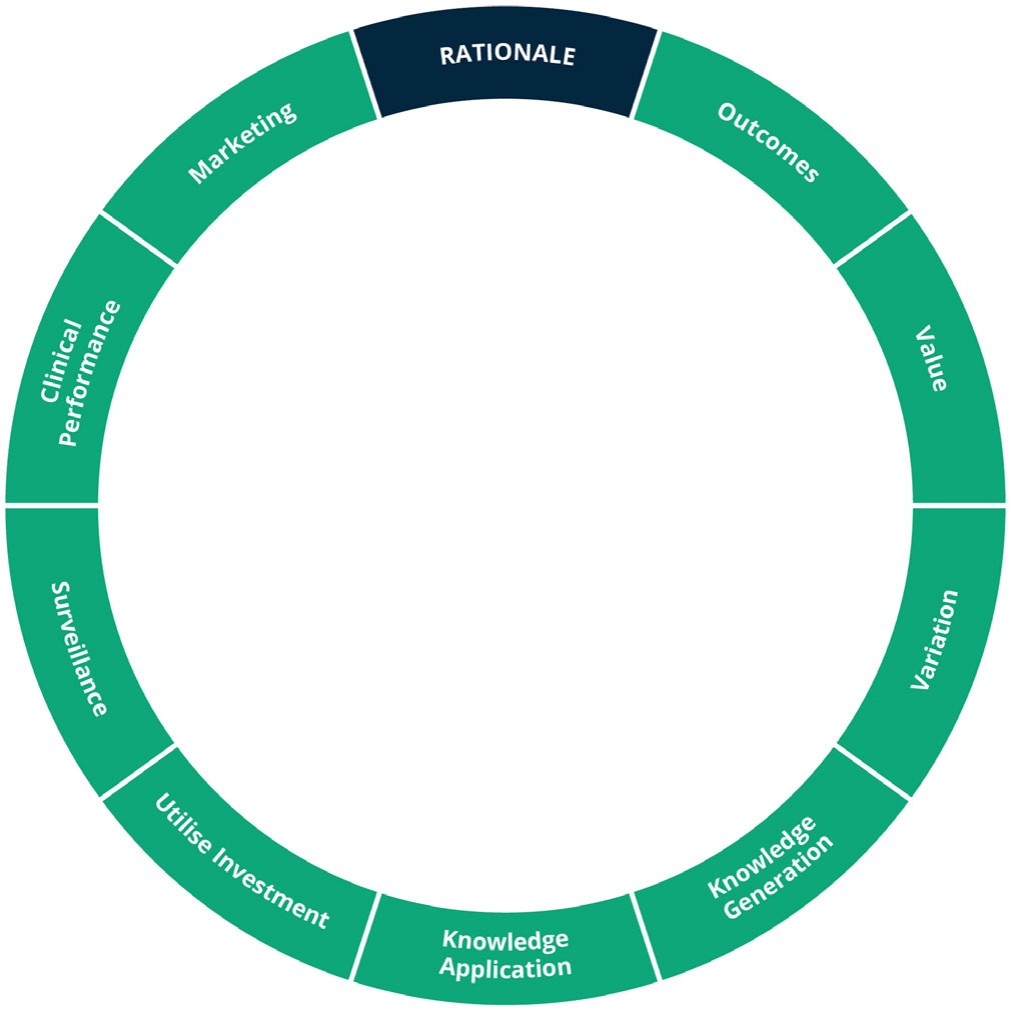
Rationale for a Learning Health System

Learning Health Systems come in many shapes and sizes. Their developers have differing objectives that influence how they are designed. Previous work^17^, ^33^ has identified a variety of different rationales for developing a Learning Health System (this is illustrated in Figure 2):

- To improve patient outcomes and experience
- To provide better value healthcare
- To identify and reduce unwarranted variation
- To generate generalisable knowledge
- To optimise the application of knowledge
- To identify and track epidemiological phenomena
- To maximise the benefits from existing technological investment
- To enhance the education, training and performance of healthcare staff
- Marketing

### 3.2 What sources of complexity exist at the system and the intervention level?

Healthcare is a complex adaptive system.^34^ It has many interacting elements, the whole is greater than the sum of the parts and outcomes are often unpredictable.^35^ As well as a clear rationale, an understanding of the complexity associated with health and Learning Health Systems is central to initiating, implementing, and sustaining Learning Health Systems in the real world. There have been several efforts to understand and manage such complexity,^2^ including the Non‐adoption, Abandonment, Scale‐up, Spread and Sustainability Framework for Health and Care Technology (NASSS). This combines 28 existing implementation frameworks and much primary research.^2^

**FIGURE 3:**
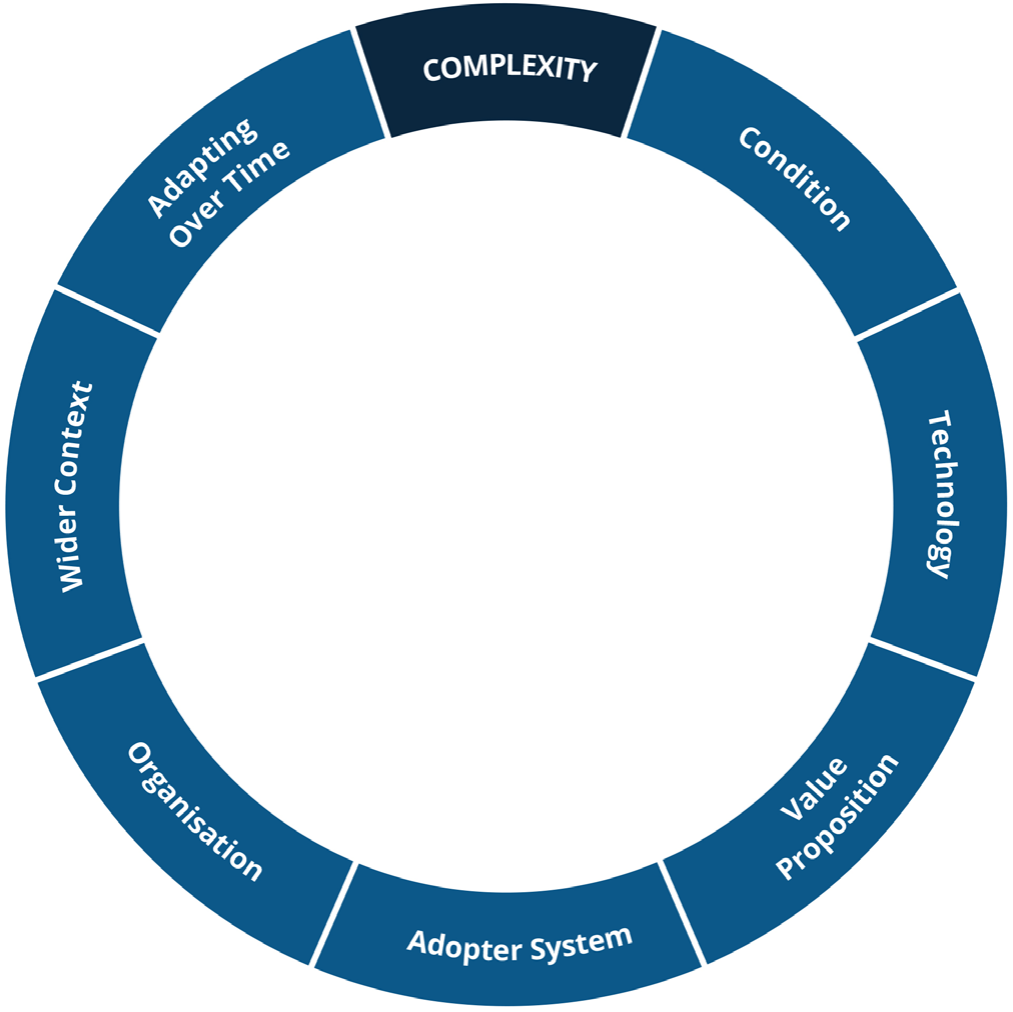
Sources of complexity in a Learning Health System (based on NASSS Framework)

The workshop on complexity in the Learning Health System, concluded that the NASSS Framework was applicable to Learning Health Systems.^36^ It has therefore been incorporated into this framework (see Figure 3).

NASSS highlights challenges to the success of an intervention across seven domains (see Figure 3). Each domain can be considered simple, complicated or complex. Too much complexity across too many domains makes the intervention more likely to fail^2^, ^37^:

1. The condition: Some illnesses are more complex than others, in terms of aetiology, prognosis, sociocultural factors, comorbidities or treatments. It is more challenging to create systems that manage complex conditions.
2. The technology: Usability, dependability, maturity and requirements to integrate with other systems can all impact success.
3. The value proposition: The intervention must deliver value to the developer, the organisation, the patient, etc. or it will not be maintained or used.
4. The adopter system: Patients and carers may abandon a system that makes unreasonable demands on them. Clinicians may resist a system that threatens their job or professional identity.
5. The organisation: The organisation's capacity for innovation and its other priorities will influence its ability to develop a Learning Health System.
6. The wider context: Laws, regulation, politics, market conditions and other events can intervene to impact a Learning Health System.
7. Adapting over time: It is essential to the long‐term success of a system. All of the other six domains are subject to change.

The NASSS Framework is available in a series of self‐assessment tools called NASSS‐CAT (Complexity Assessment Tool).^37^ They can be applied to an entire Learning Health System or to individual interventions within a Learning Health System and are useful at the design, implementation and evaluation stages.^36^ They also include advice on reducing and responding to complexity.

### 3.3 What strategic approaches to change do we need to consider?

**FIGURE 4:**
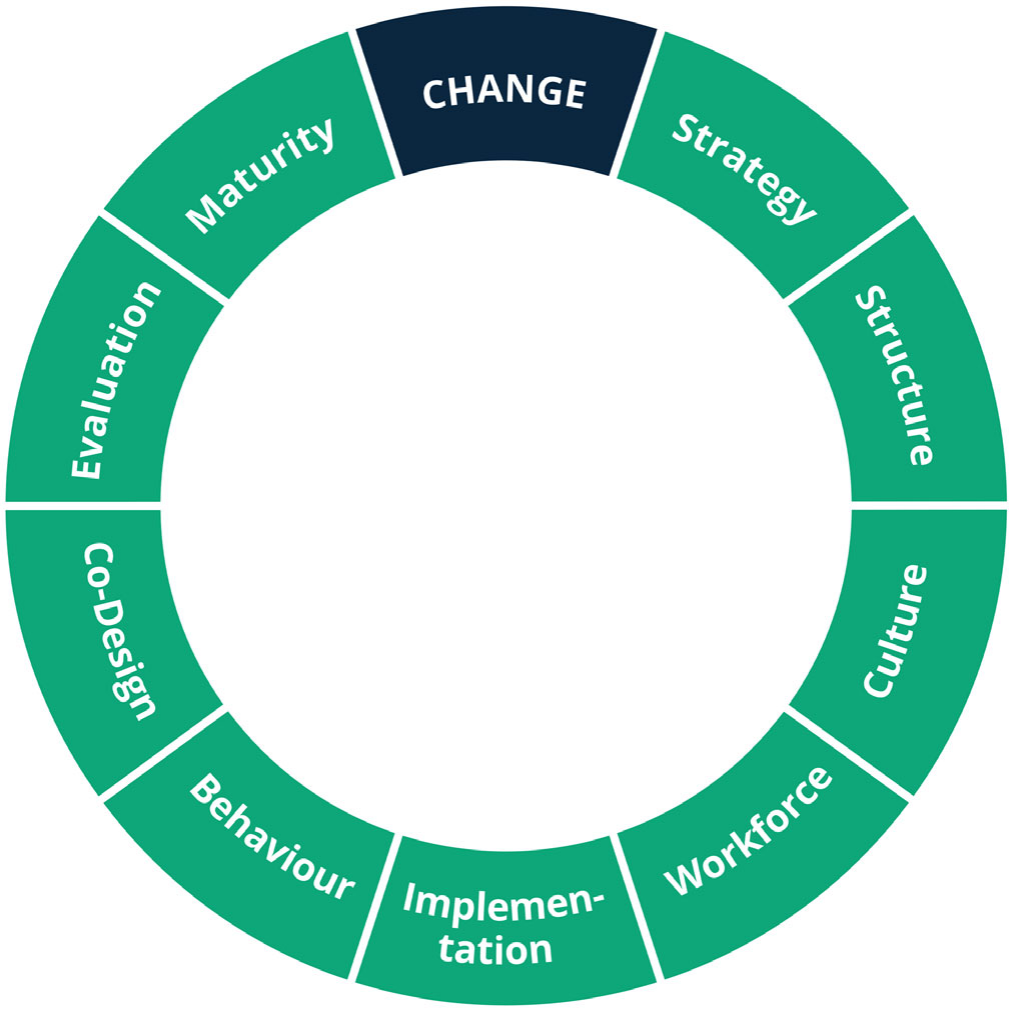
Strategic approaches to change in a Learning Health System

Continuous improvement within a Learning Health System requires change. The earlier report,^17^ workshops and the literature, suggest a number of areas in which strategic approaches to change are required (see Figure 4).

*Strategy*: A Learning Health System requires a strategy and must be part of the broader strategy of its host organisation(s). The strategy is a set of inter‐related choices on how the organisation will deliver value.^38^ It can be an explicit document, outlining goals, priorities and actions, or it can be “a pattern in a stream of decisions” ‐ a strategic direction.^39^ The stakeholders must co‐design the strategy. There are many guides and tools that can help with this.^40^, ^41^, ^42^, ^43^

*Organisational Structure*: People and resources must be organised to deliver the strategy. This is sometimes called a target operating model (TOM) or an organisational architecture^44^ and has been explicitly addressed in various Learning Health Systems.^45^ Care is required to ensure that this approach does not become overly mechanistic, making unrealistic assumptions about the future.

Other approaches including, rapid prototyping, microstrategies, safe‐to‐fail experimentation^46^ and actor‐oriented learning networks,^47^ have been employed to more effectively meet the needs of Learning Health Systems.^48^ Actor‐oriented learning networks have been employed by ImproveCareNow, to create a Learning Health System for Inflammatory Bowel Disease^49^ and have since been spread more widely.^14^

*Culture*: It has long been recognised as central to a Learning Health System.^50^ The organisation must be open to learning, innovation, sharing, research, etc.^51^ Culture can be difficult to describe but can be thought of in 3 layers^52^:

- Visible manifestations: Professional roles, physical layout of facilities, pathways of care, etc.
- Shared ways of thinking: Values and beliefs that justify the visible manifestations.
- Deeper shared assumptions: Unconscious underpinnings of daily practice.

These layers are learned and shaped through experience and distinct sub‐cultures can develop, such as among clinicians and managers.^52^ There are quantitative and narrative‐based tools for assessing culture, but none is applicable in all situations.^53^, ^54^ Influencing culture is even more challenging, however, key targets and tools have been identified, with a focus on: vision, values, goals, compassion, learning, innovation, teamwork and collective leadership.^55^

*Workforce*: Developing a Learning Health System is a multidisciplinary endeavour. Patients, carers, clinicians, researchers, designers, strategists, analysts and other professionals have a role to play, depending on the particular implementation. The team could be made up from a mix of employed, contractors and consultants. Other staff may be employed by suppliers and platform providers. Leadership is especially important within such complicated organisational structures.^56^

As well as traditional responsibilities, clinicians will also need new skills and competencies:

- Awareness of how the data that they record will be used.^57^
- Interpreting the results of data analytics.^58^, ^59^, ^60^
- Confidence with research as part of the clinical role.^58^
- Ability and willingness to use data from non‐healthcare sources.^57^
- Ability to use rapid and voluminous feedback on their own practice.^61^

Many of these are represented in the Faculty of Clinical Informatics Competency Framework^24^ and take years to develop.

*Implementation Strategy*: A Learning Health System will often require an integrated package of evidence‐based interventions, designed to overcome barriers to successful implementation. This is known as an implementation strategy.^62^

*Behaviour Change*: A successful Learning Health System will require individuals and organisations to change what they are doing. This often requires purposeful intervention. There are many models that can be employed to guide this process.^63^ These often involve understanding what drives a given behaviour and then selecting techniques that can influence those drivers.

*Co‐design*: The complexity of a Learning Health System can only be understood by involving the stakeholders in its ongoing development.^64^, ^65^ Design principles have been developed,^66^ based on ISO 9241‐210, the international standard in human‐centred design for interactive systems.^67^ Libraries of open‐source co‐design tools are available.^68^, ^69^, ^70^ The choice of tools can be influenced by, the task, the type of knowledge sought and by the participants,^71^ but there is limited evidence on how to choose the most appropriate tools.^71^, ^72^ It can be helpful to employ an overarching co‐design framework,^73^, ^74^ that offers a step‐by‐step guide. Crucially, it must not be a one‐off process, but rather a continuous part of a Learning Health System.

*Appraisal and Evaluation*: Guidance is available on appraising whether or not to build a Learning Health System,^75^ but, in practice, the decision can be reached by a multitude of formal and informal means. It might be driven by top‐down directive or bottom‐up interest. Pilots can be useful in testing hypothesised impacts.^76^

Once implemented, it is important to evaluate how effective, safe and cost effective a Learning Health System is. This allows an organisation to change direction and can enable others to avoid the same mistakes. There is guidance on evaluating complex systems,^77^ but Learning Health Systems can present particular challenges. A range of quantitative and qualitative methods might be appropriate (see Table 2). The mix will depend on the situation. Continuous improvement cycles, such as Plan‐Do‐Study‐Act, might be more feasible in some situations.^76^

**TABLE 2:** Example analytic approaches within a Learning Health System^16^

Analytic approach	Ideal use	Examples
Randomised trials	The most powerful knowledge generation methods in determining whether two treatments differ	Randomised controlled trials,^78^ A/B testing^79^
Quasi‐experimental analyses	When randomisation is challenging. For example, when a new policy is introduced nationally	Difference‐in‐differences analysis, interrupted time series,^80^ regression discontinuity,^81^ causal inference,^82^ artificial control groups,^83^ statistical process control^84^
Artificial Intelligence	Where large datasets exist, promising uses include, identifying undiagnosed disease, identifying new disease subtypes, predicting events or selecting treatments^85^	Deep learning,^86^ Machine learning,^87^ Penalised regression^88^
Descriptive statistics	When patterns can be identified without complex statistical techniques	Visualisation, process and value stream mapping^89^
Qualitative methods	When it is necessary to make sense of complex sociotechnical environments and to understand the why and how as well as the quantitative what	Interviews, focus groups, surveys and ethnography^90^

*Maturity*: In addition to evaluating outcomes, it is important to understand the degree to which processes within the Learning Health System are able to achieve those outcomes in a predictable way.^91^ There are widely used maturity models for considering technical infrastructure^92^ and there are process maturity assessment tools aimed at the complex, constantly changing sociotechnical aspects of a Learning Health System.^91^

### 3.4 What technical building blocks will we need?

Once an organisation(s) understands why it is developing a Learning Health System and begins to appreciate the complexity and how change will be delivered, it can develop the technical building blocks required. Most Learning Health Systems build upon existing legacy systems. Locally, these legacy systems include electronic health records, data warehouses, decision support systems, learning communities and other sociotechnical systems. Nationally, these can include data sets, registries, guideline and policy development forums.

**FIGURE 5:**
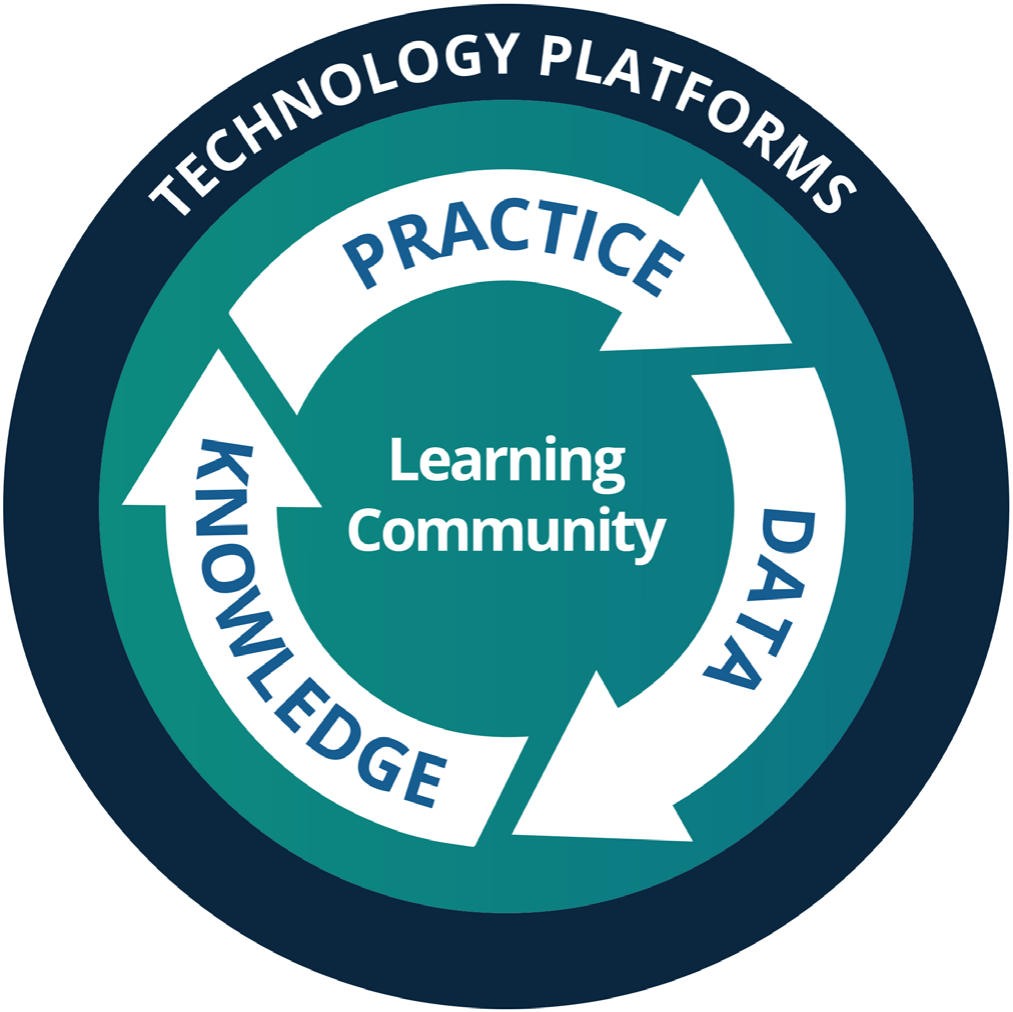
Technical building blocks of a Learning Health System

An early analysis of these systems, including strengths, gaps and opportunities for development, is therefore often a prerequisite to developing the technical building blocks. Resources are always limited and this analysis can help to prioritise where they can have most impact. Friedman and colleagues^9^ have developed a generally accepted model, illustrating the core activities of a Learning Health System. This has been adapted to organise the technical building blocks within the new framework (Figure 5).

### 3.5 Learning communities

A learning community is a group of stakeholders (which may include patients, clinicians, managers, administrators, researchers, technical staff and others) who come together to achieve collective improvement. They are the centre of a Learning Health System. The learning community should drive the development and operation of a Learning Health System. They should implement a process for deciding what is to be studied and will also generate knowledge through reflection and shared experience. Such human communities are well placed to make sense of the complexity associated with a Learning Health System. The learning community may develop from an existing forum and there are helpful guides to operationalising a learning community,^93^, ^94^ but they should be tailored to each Learning Health System.

### 3.6 Practice to data

Once the learning community has decided what to study, the next step is to collect data that represents what is going on within the system. This requires several technical building blocks:

- *Data capture*: Often from existing sources, such as the electronic health record, laboratories, remote monitoring tools or from technology (such as smartphones, wearables), social media, surveys, interviews, etc.^17^, ^58^, ^95^, ^96^, ^97^, ^98^
- *Data Quality and Provenance*: Routine data is collected as a by‐product of care, often without thought to their secondary uses. Quality can be variable. In order to rely on such data, it is important to consider the history of the data items and how they came to be in their current state.^99^
- *Data Storage and Access*: Multi‐organisation Learning Health Systems must agree on whether data will be submitted to a centralised data store, such as England's Hospital Episode Statistics,^100^ or held locally in a distributed network, such as the FDA Sentinel Programme.^101^ The former offers simplicity and economies of scale, while the later offers local control. Access to data can be disseminated to trusted parties (bringing data to the analysis) or users can analyse the data within a secure trusted research environment (bringing the analysis to the data).
- *Information Governance*: Trust in a Learning Health System depends on data being collected, stored and shared within a strong and ethical framework.^102^ In the United Kingdom and European Union, this is unpinned by the General Data Protection Regulation.^103^ In the United States, data sharing is regulated by the Health Insurance Portability Act.^104^
- *Interoperability*: Learning Health Systems are often a network of networks that must be able to work with each other. This requires standards around, collection, the terminologies and classifications used, as well as the structure, transport and security of data.^17^, ^28^, ^105^ With longitudinal data, standards for its representation can change over time, requiring complex mapping.

### 3.7 Data to knowledge

Data is of little value unless it can be turned into knowledge. Knowledge is the insight produced by analysing data and interpreting the product of that analysis. This could include a guideline, predictive model, calculator, etc. Organisations often collect huge amounts of data but fail to turn it into generalisable knowledge.^28^ Within a Learning Health System, knowledge can be generated by many statistical and deliberative methods, such as those shown in Table 2. These are described in more detail elsewhere.^16^

Locally generated knowledge must be integrated with externally produced knowledge such as peer reviewed research or service evaluations conducted elsewhere. The objectives of the Learning Health System and the resources and infrastructure available will help to determine the methods and data which are most appropriate. This process should be led by the learning community.

### 3.8 Knowledge to practice

To improve health and care, it is not enough to generate knowledge. It must be applied. Traditionally, knowledge has been represented in books, journal articles, guidelines and protocols. It has been passed on through publications, education, training and word of mouth, before being unevenly applied.

These methods will continue to be essential within a Learning Health System, but there have also been efforts to standardise the representation of knowledge,^106^, ^107^ so that it can be delivered through decision support systems or used to assess whether real‐world care conforms to the evidence. This may greatly reduce the time taken for knowledge to be applied.

There are many challenges to mobilising knowledge within a Learning Health System^23^, ^30^, ^31^, ^95^:

- Size of the task: The body of medical knowledge is huge.
- Standards: Are required to make medical knowledge interoperable.
- Ambiguity: Unlike traditional guidelines, computable guidelines require all background knowledge and common‐sense assumptions to be made explicit.
- Inference: knowledge must be combined with data about the specific situation or patient to produce actionable outputs. This becomes even more challenging when there are conflicting knowledge sources, multiple valid interventions, multi‐morbidity, polypharmacy, etc.
- Outcomes: Must be tracked to understand their magnitude and to who they accrue.
- Safety: When such systems malfunction, large numbers of staff and patients can be affected.^108^ Decision support systems may be considered medical devices under some regulatory environments, such as the United Kingdom,^109^, ^110^, ^111^, ^112^ and therefore require extensive development and validation.
- Professionalism: Including clinical problem solving, craft skills and compassion, cannot be entirely replaced by computable knowledge.

For these reasons, progress on mobilising computable knowledge has often been slow.

In England, The National Institute for Health and Care Excellence (NICE) is planning a staged progression toward the goal of mobilising computable knowledge. NICE provides evidence‐based guidance to England's National Health System. They have assessed progress against the Agency for Healthcare Research and Quality's four levels of knowledge: narrative, semi‐structured, structured and executable.^27^, ^113^ From this they have concluded that executable guidelines cannot be practically or safely reverse engineered from existing narrative guidelines. A new co‐production model of guideline development will be required, with different processes, methods, technologies and skills.^27^

### 3.9 Platforms

Much of the infrastructure required to build a Learning Health System is common across organisations. This can include IT, Electronic Record Systems, data transport, storage and processing, decision support and even governance arrangements and organisational structures. The development of platforms that provide this infrastructure as a service, is making it feasible and affordable for even small organisations to develop Learning Health Systems.^95^ The Mayo Clinic is pursuing this approach through its 10‐year Platform Strategic and Operating Plan.^114^ They are purchasing platform services from other organisations, such as Google Cloud. They also hope to build platform services that may eventually be offered to other healthcare providers. These include a data platform for analytics, a virtual care platform for video consultations and remote monitoring and a remote diagnostic and management platform.^95^

The workshop on Platforms in the Learning Health System, agreed that “Platform Thinking” may reduce the technical demands on an organisation and enable economies of scale, but it requires business transformation and more collaboration with and trust for commercial and other partners.^95^

## 4 DISCUSSION

The elements of the framework, outlined in the previous section (Figures 2, 3, 4 and 5), have been developed through the analysis of interviews, workshops and purposeful literature reviews. They can be combined to create the overarching framework shown in Figure 1. Each ring represents elements of the answer to one of the questions in Box 1. The questions and thus the rings are related, but there is no direct mapping from the elements of one ring to those of the next.

The intention has been to create a framework that helps those wishing to understand, design, analyse or evaluate, real‐world Learning Health Systems, by highlighting elements that should not be overlooked. It is not intended to be a step‐by‐step, how‐to guide. The complexity of a Learning Health System would render such a guide ineffective because of the presence of non‐linear, unpredictable and constantly changing relationships within and between the elements, as described in the results section on complexity.

For the same reason, the framework does not explicitly position patients or clinicians, even though they are the most important actors in a Learning Health System. Instead, their presence is to be found in every ring of the framework, including within the learning community, at its centre.

In the design process, Figure 1 represents a framework for organising the key technical building blocks and platforms that will be required to deliver the aims of a proposed Learning Health System. It also highlights the potential sources of complexity and the strategic interventions that will be required to manage that complexity.

Organisations will often want to evolve their existing health system towards the goal of becoming a Learning Health System. In this scenario, the existing system can be mapped onto the framework, thus highlighting areas of strength, weakness or uncertainty. These can then be addressed systematically, using the tools identified.

Likewise, an organisation that has already embarked on a Learning Health System journey, can evaluate their progress in each element of the framework.

Learning Health Systems have been described as exhibiting fractal‐like properties,^115^ meaning that the actions required to facilitate them are similar at whatever scale ‐ local, regional, national or international. The framework shown in Figure 1 shares that property. Each of the elements are relevant, regardless of the scale at which a Learning Health System is being developed.

### 4.1 Strengths, limitations and future work

This framework was developed based on the insights of a wide, but necessarily finite, group of subject experts and a series of purposeful literature reviews. The topics identified spanned 30 areas, each of which is a separate and evolving area of study. It was beyond the scope of this work to conduct systematic literature reviews on each of these areas. It is therefore possible that important insights have been missed or excluded due to space or capacity constraints.

To overcome this limitation, The Learning Healthcare Project have published a longer report, containing links to many additional resources.^16^ Additionally, a collaboration of academics from Australia, the Netherlands, the United Kingdom and the United States has been formed to develop an online toolkit, based on the framework. Eventually, it should be possible for users around the world to add helpful Learning Health System tools. Analysis of these tools, patterns of usage within the toolkit and qualitative feedback will be used to evolve the framework's content and visual appearance, ensuring that it remains up‐to‐date, useful and becomes increasingly representative.

This paper began by identifying other frameworks and reasons why they might not have been adopted. It remains to be seen whether this framework will be any more widely adopted. It has been seven months since it was first published. Since then, the project website has had 9023 individual users from 149 countries (US 30%, UK 24%, Canada 10%, Australia 5% and China 4%). The lead author has been invited to present the framework to 16 national and regional forums. It has been used at NHS Digital to conduct a desk‐based training exercise on Learning Health Systems. Health Education England have embarked on a process to review their activity against the framework. The lead author has also applied the framework to assess the potential for a national Learning Health System initiative in England.

Early feedback from the above activities has resulted in some evolution of the framework, between the initial publication and this paper:

- The importance of Learning Communities has been elevated, moving them to the centre of Figure 1.
- It has been recognised that while Figure 1 is a useful visual checklist, it is not easily interpreted without detailed textual description.
- The framework has therefore become more focused on the four questions in Box 1.
- Figure 1 has been removed from the prototype toolkit and replaced with the four questions.
- The four questions have been reordered.
- The third question has been refocused on change, rather than just strategy.

This evolution is expected to continue and a governance system will be established to coordinate regular updates.

## 5 CONCLUSION

Interest in Learning Health Systems has grown since the term was first coined in 2007.^33^ This has created demand for a scalable framework to guide their development. This paper outlines such a framework, covering the rationale for a Learning Health System, sources of complexity, approaches to change and its technical building blocks.

It is hoped that this framework will aid those designing, analysing or evaluating Learning Health Systems. The framework itself is not set in stone, but will be updated over time, based on usage and feedback from the community.

## CONFLICT OF INTEREST

Prof Luke Vale has no conflicts of interest to report. Dr Tom Foley has been commissioned by Health Education England to apply the framework to their efforts to become part of a national Learning Health System.
